# Oxidative Stress-Related Susceptibility to Aneurysm in Marfan’s Syndrome

**DOI:** 10.3390/biomedicines9091171

**Published:** 2021-09-06

**Authors:** Jacek Rysz, Anna Gluba-Brzózka, Robert Rokicki, Beata Franczyk

**Affiliations:** 1 Department of Nephrology, Hypertension and Family Medicine, Medical University of Lodz, 90-549 Lodz, Poland; jacek.rysz@umed.lodz.pl (J.R.); beata.franczyk-skora@umed.lodz.pl (B.F.); 2 Clinic of Hand Surgery, Medical University of Lodz, 90-549 Lodz, Poland; robert.rokicki@umed.lodz.pl

**Keywords:** Marfan’s syndrome, aortic aneurysm, oxidative stress

## Abstract

The involvement of highly reactive oxygen-derived free radicals (ROS) in the genesis and progression of various cardiovascular diseases, including arrhythmias, aortic dilatation, aortic dissection, left ventricular hypertrophy, coronary arterial disease and congestive heart failure, is well-established. It has also been suggested that ROS may play a role in aortic aneurysm formation in patients with Marfan’s syndrome (MFS). This syndrome is a multisystem disorder with manifestations including cardiovascular, skeletal, pulmonary and ocular systems, however, aortic aneurysm and dissection are still the most life-threatening manifestations of MFS. In this review, we will concentrate on the impact of oxidative stress on aneurysm formation in patients with MFS as well as on possible beneficial effects of some agents with antioxidant properties. Mechanisms responsible for oxidative stress in the MFS model involve a decreased expression of superoxide dismutase (SOD) as well as enhanced expression of NAD(P)H oxidase, inducible nitric oxide synthase (iNOS) and xanthine oxidase. The results of studies have indicated that reactive oxygen species may be involved in smooth muscle cell phenotype switching and apoptosis as well as matrix metalloproteinase activation, resulting in extracellular matrix (ECM) remodeling. The progression of the thoracic aortic aneurysm was suggested to be associated with markedly impaired aortic contractile function and decreased nitric oxide-mediated endothelial-dependent relaxation.

## 1. Introduction

The roles of oxidative stress and antioxidants have been widely described in numerous articles. The involvement of highly reactive oxygen-derived free radicals (ROS) in the genesis and progression of various cardiovascular diseases, including arrhythmias, aortic dilatation, aortic dissection, left ventricular hypertrophy, coronary arterial disease and congestive heart failure, is well-established [1,2]. The levels of free radicals are controlled by the activity of antioxidants. At low concentrations, ROS control cell signaling, proliferation and vascular tone, however, if their concentration becomes elevated, they start to trigger the development of many diseases [3,4]. The imbalance between the generation of reactive oxygen species and the antioxidant capacity of the biological system results in oxidative stress [5,6]. It was found that all cell types present in the vasculature have enzymes capable of ROS generation, such as nicotinamide adenine dinucleotide phosphate NADPH oxidase (Nox) and xanthine oxidase (XO) [7,8]. ROS is associated with enhanced oxidation of low-density lipoproteins (LDL), cholesterol-derived species as well as modifications of proteins, all stimulating the formation of foam cell and atherosclerotic plaques’ development in arteries [9]. It was also found that oxidative stress and vascular dysfunction play an important role in the development of aortic pathologies associated with the loss of contractile function and endothelium-mediated relaxation [10]. Moreover, these factors may modify the mechanical properties of the vessel, resulting in the formation of a pseudoaneurysm or aneurysm [11]. Certainly, molecular oxygen is not the only perpetrating agent since aortic damage seems to be multifactorial, however, surely, it plays a critical role in vascular tonicity, cardiac contractility, etc. [5,10].

The results of studies have indicated that the intake of antioxidants is associated with protection against cardiovascular diseases. Antioxidant vitamins have been demonstrated to regulate endothelial nitric oxide levels, and inhibit lipid peroxidation, cardiovascular inflammation and platelet aggregation to prevent endothelial dysfunction [12]. However, it was also suggested that excessive consumption of antioxidants may be related to potential toxicity [12].

In this review, we will concentrate on the impact of oxidative stress on aneurysm formation in patients with Marfan’s syndrome as well as on the possible beneficial effects of some agents with antioxidant properties.

## 2. Marfan’s Syndrome (MFS)

Marfan’s syndrome is a genetic disorder affecting connective tissue. The prevalence of classic Marfan’s syndrome was estimated to be about 2–3 per 10,000 individuals, however, it may not be accurate since there are many factors (common outward manifestations, the fact that phenotype becomes more apparent with increasing age, family history of Marfan’s syndrome is not always present) which can contribute to the underestimation of this disease incidence [13]. Characteristic features of this disorder, such as tall stature with dolichostenomelia, make it slightly more frequently occurring in certain types of athletes, including basketball and volleyball players [14].

### 2.1. Cause of Marfan’s Syndrome

Marfan’s syndrome is inherited in an autosomal dominant manner. This disorder has been found to be caused by at least 1847 different *fibrillin-1 (FBN1)* gene mutations [15]. The consequence of these mutations involves the impairment of fibrillin metabolism [16]. The *fibrillin-1* gene, located on chromosome 15q21.1, contains 65 exons and it encodes a 350 kDa glycoprotein, which is highly conserved among different species [13]. The results of studies indicated that most mutations appear within 47 tandemly repeated epidermal growth factor-like domains. Many of them damage one of the six predictably spaced cysteine residues, thus leading to boosted cleavage and proteolytic degradation [17,18]. According to studies, half-normal synthesis of normal protein (haploinsufficiency), rather than the production of the mutant protein, could be of key importance to reach the threshold loss of fibrillin-1 function required for clinical expression of the disorder [13]. Fibrillin-1 is a key component of extracellular microfibril, which provides a scaffold for elastic fiber formation and maturation [19,20]. Therefore, mutant fibrillin-1 impairs microfibril formation, resulting in medial degeneration, which is mirrored by poor alignment of elastin filaments, altered organization of lamellar units, the accumulation of proteoglycan and vascular smooth muscle cell (VSMC) death [21]. Changes in fibrillin-1 disturb elastic fibers’ integrity within the endothelial layer, leading to impaired endothelial permeability observed in patients with Marfan’s syndrome [22]. The development of such modifications disrupts the aortic wall, making the aorta vulnerable to hemodynamic injury [23,24]. Apart from mutations in the *fibrillin-1* gene, also changes within transforming growth factor β receptor 2 (TGF-βR2) and TGF-βR1, which regulate extracellular matrix synthesis and homeostasis, can cause Marfan’s syndrome [25,26,27]. According to estimations, up to 5% of MFS patients are carriers of the mutation in the *TGF-βR2* gene [28]. These alterations were found to be associated with aortic dilation [29]. In 25% of cases, Marfan’s syndrome is caused by de-novo mutations. Missense mutations account for 60% of alterations. Extensive linkage and comprehensive mutation analyses have demonstrated that there is no locus heterogeneity for the classic Marfan’s phenotype [30,31]

### 2.2. Marfan’s Syndrome Manifestations

Marfan’s syndrome is a multisystem disorder with manifestations including cardiovascular, skeletal, pulmonary and ocular systems [13]. Cardiovascular manifestations of Marfan’s syndrome can be divided into those affecting the heart, especially atrioventricular valves and leading to prolapse of either the mitral or tricuspid valves or both atrioventricular valves, and those affecting the vasculature, the latter being the topic of this review [32]. As a result of these alterations, patients with Marfan’s syndrome may suffer from congestive heart failure, dilated cardiomyopathy, pulmonary hypertension, calcification of the mitral annulus, aortic valve dysfunction, ventricular dysrhythmia and atrial fibrillation [33,34]. However, aortic aneurysm and dissection are still the most life-threatening manifestations of Marfan’s syndrome [13]. The most common localization of aneurysm in MFS is the aortic root—descending thoracic aortic aneurysm (TAA) occurs less commonly, while abdominal aortic aneurysm (AAA) is rarely reported in these patients [35]. Despite the fact that dilatation at the sinuses of Valsalva develops even in utero in severe cases, the aortic size in some patients with this disease never reaches the point at which it will require surgical intervention. Severe aortic valve insufficiency sometimes complicates the aortic dissection. The involvement of carotid arteries can lead to cerebrovascular injury and neurological sequelae, while the involvement of coronary arteries may result in myocardial infarction or sudden cardiac death, usually related to rupture into the pericardial sac with subsequent pericardial tamponade. MFS patients’ lifespan is often shortened, and the predominant causes of deaths, diagnosed in 90% of cases, include cardiovascular diseases (aortic dissection, congestive heart failure or cardiac valve disease).

## 3. Oxidative Stress

The imbalance between the generation of pro-oxidative species and the antioxidants results in changes in redox state and oxidative stress [5,6]. ROS are released as a by-product of natural oxygen metabolism and they are involved in both normal cell signaling and homoeostasis [36,37]. ROS and reactive nitrogen species (RNS) are generated via different pathways, such as mitochondrial xanthine oxidase and NADPH oxidase. Oxidative stress is involved in the pathogenesis of various cardiovascular diseases. It was demonstrated to cause endothelial dysfunction via direct and indirect mechanisms, including the scavenging of nitric oxide (NO) by superoxide [10]. NO participates in the regulation of smooth muscle contractility, and thus, it controls vascular smooth muscle tone and mechanical properties. Due to the fact that the rate of binding superoxide radicals to NO is three times higher than to superoxide dismutase (SOD), the presence of excess superoxide results in enhanced NO degradation [8]. Furthermore, peroxynitrite, which is formed in the reaction between superoxide and NO, could cause the uncoupling of eNOS via the oxidization of tetrahydrobiopterin and subsequent decrease in NO production [7]. The presence of oxidative stress results in endothelial dysfunction and decreased production of NO, which translate into vascular stiffness, reduced distensibility and aortic complications.

Oxidative stress in the vessel wall may be associated with the activity of Nox. NADPH oxidase is the predominant contributor of superoxide anions in the vasculature as it catalyzes the reduction of oxygen with electrons from NADH or NADPH [38,39]. The exact mechanism of the impact of reactive oxygen species on smooth muscle cell (SMC) contractility remains unknown, however, ROS was suggested to adversely influence calcium signaling in both vascular endothelial and smooth muscle cells, which could potentially lead to the alteration of vascular reactivity [40,41,42]. Additionally, decreased neutralization of superoxide in the vasculature, possibly due to the downregulation of SOD-1 and SOD-2, leads to oxidative stress [8]. Isoforms of the SOD enzyme play an essential role within the vascular wall since they reduce superoxide levels, thereby preventing endothelial injury [5,43]. Cu-Zn-SOD is the predominant isoform, relatively highly abundant in all cell types. Genetically modified mice with the overexpression of Cu-Zn-SOD were demonstrated to be protected against vascular dysfunction [44]. Extracellular Mn-SOD is another isoform [5]. In a normal state, Mn-SOD is the first line of defense against oxidative stress, however, its expression may be modified in the presence of oxidative stress [45]. The results of studies revealed that enhanced expression of Mn-SOD could be triggered by proinflammatory cytokines and lipopolysaccharides (LPS)-mediated inflammation in vascular tissue [46]. In turn, the inactivation of this enzyme is associated with the presence of high concentrations of peroxynitrite [47]. It was implied that the elimination of superoxide via SOD and catalase activity may improve calcium signaling, restoring contractile responses. The treatment with SOD was demonstrated to reverse the hypersensitivity of the arteries in diabetic and hypertensive animal models [48]. Catalase (CAT) is another significant antioxidant enzyme, which uses two H_2_O_2_ molecules to break them into O_2_^−^ [5]. It has been revealed that the overexpression of CAT prevents the stimulation of ROS [49].

## 4. Aortic Aneurysm Formation in Marfan’s Syndrome and the Role of Oxidative Stress

### 4.1. Aortic Aneurysm Formation in Marfan’s Syndrome

The mechanisms behind vascular pathology involve the creation of extracellular matrix (ECM) microfibrils as structurally abnormal as a result of the insufficient formation of fibrillin-1 polymers, leading to the detachment of VSMC from the elastic laminae, followed by enhanced VSMC apoptosis, the release of matrix metalloproteinases (MMPs) and finally, weakened and disordered elastic fiber construction in aortic tissue [50,51,52,53]. The disturbed microfibril network linking elastic lamellae with adjacent VSMC impairs the integrity of the aortic wall [54]. The decrease in total fibrillin-1 synthesis below a specific threshold is associated with connective tissue weakness [55]. The results of animal studies confirm markedly lower elastin orientation indices in MFS ascending the aorta, which translate into decreased resistance to strain and impaired load-bearing capacity [56,57]. According to Schwaerzer et al. [58], mechanisms underlying thoracic aortic pathology associated with constitutive serine/threonine protein kinase (PKG1) activation involve activation of c-Jun N-terminal kinase (JNK), SMC apoptosis and MMP-2 activation, increased TGF-β expression and NADPH Oxidase 4 (NOX4) activation. The presence of diminished or defective forms of fibrillin-1 were demonstrated to be associated with disturbed matrix sequestration of the large latent complex (LLC), with consequent excessive activation and signaling of TGF-β [59]. The aortic pathology is also related to augmented *TGF-β* expression, especially in the late stages of aneurysm progression [58,60]. Enhanced TGF-β signaling can lead to the activation of the non-canonical p38 mitogen-activated kinase (MAPK/ERK) pathway in VSMC, the subsequent increase in the level of plasminogen activators, the upregulation of MMP transcription and finally ECM degradation [53,61,62]. The confirmation of the involvement of this mechanism was obtained from a study indicating that the development of aortic root aneurysm was inhibited in MFS mouse models treated with anti-TGF-β antibodies [63]. Activation of the MAPK/ERK pathway is also associated with VSMC proliferation, fibrosis, boosted expression of MMP-2 and MMP-9 and decreased apoptosis, resulting from the binding of angiotensin II (AngII) to angiotensin II receptor type 1 (AT1) receptors within the aortic wall [64]. Indeed, the infusion of AngII was demonstrated to stimulate the development of ascending aortic aneurysms (AA) in wild-type mice [65]. In turn, endothelial dysfunction in MFS results from impaired endothelium-dependent relaxation in the thoracic aorta, probably due to the downregulation of Akt/eNOS-induced production of NO [22]. Therefore, it appears that NO production induced by shear stress may be a vital contributor to the pathogenesis of thoracic aneurysm manifestations in MFS. The activation of JNK is associated with upregulating NOX4 and subsequent enhancement of JNK activation. Both JNK activation and the presence of oxidative stress can induce SMC apoptosis as well as MMP-2 activation, resulting in elastin fiber degradation. The involvement of increased reactive oxygen species and JNK activation have been demonstrated in various types of thoracic aortic aneurysm and dissection (TAAD) and abdominal aortic aneurysms [10,66,67]. In TAAD, the upregulation of NOX4 was observed. Such upregulation translated into higher hypoxia-inducible factor 1 (HIF-1) and vascular endothelial growth factor A (VEGF-A) expression and dysregulated HIF-1α/VEGF signaling was associated with aortic aneurysm progression [68,69].

### 4.2. Role of Oxidative Stress in Aortic Aneurysm Formation

Many recent reports indicated increased ROS levels within the aortic root aneurysm wall in patients with Marfan’s syndrome as well as in murine specimens [10,70]. It has been suggested that reactive oxygen species may be involved in smooth muscle cell (SMC) phenotype switching and apoptosis and matrix metalloproteinase activation, resulting in ECM remodeling [71,72,73]. Pathological effects associated with abundant ROS also include the stimulation of proinflammatory genes [74]. The presence of enhanced oxidative stress in Marfan’s syndrome was confirmed by the finding of increased levels of isoprostane 8-epi-PGF2α in the plasma and aortic homogenate of Marfan’s mice compared with the control [75,76]. Additionally, Fiorillo et al. [77] provided evidence of plasmatic signs of oxidative stress in MFS. Moreover, they suggested that in MFS, the intensity of oxidative stress could be a marker of the clinical severity and the number of organs/systems affected. Oxidative stress has been demonstrated to disrupt mechano-signaling within the aortic wall, stimulate pathological switching of VSMCs phenotype and their apoptosis and enhance the expression of ECM-degrading matrix metalloproteinases (MMP) [78,79]. All the aforementioned processes induce aortic wall degeneration and thoracic aortic aneurysm formation [80]. Branchetti et al. [81] confirmed the presence of increased peak wall stress in TAA patients, which correlated with ROS accumulation and switching towards pathological synthetic VSMC populations. Recent studies have demonstrated deficiency of total antioxidant capacity (TAC) in plasma of MFS patients, which correlates with clinical severity in this disease [77]. The results of studies performed on animal models of MFS indicated impaired aortic contraction and relaxation of the aorta in vivo, as well as enhanced lipid peroxidation due to decreased levels of ROS-scavenging of antioxidant and increased expression of pro-oxidant enzymes [10]. Interestingly, they found that higher production of ROS was limited solely to the aneurysmal site [82]. Moreover, the restoration of normal redox homeostasis with the use of ROS inhibition or antioxidant supplementation was found to ameliorate vasomotor function and decrease aneurysmal dilatation [79]. According to many studies, mechanisms responsible for oxidative stress in an MFS mouse model involve decreased expression of SOD as well enhanced expression of NAD(P)H oxidase, inducible nitric oxide synthase (iNOS) and xanthine oxidase [77]. Higher plasma homocysteine (Hcy) levels observed in patients with Marfan’s syndrome and severe cardiovascular involvement could be responsible for higher ROS production by NADPH oxidase [64]. NADPH oxidase was found to be the predominant source of ROS in AAA, as reflected by its higher expression in the human aorta from patients with AAA [83]. The NADPH oxidase system comprises a family of NOX homologs: NOX1, NOX2, NOX3, NOX4, NOX5, dual oxidase 1 (DUOX1) and DUOX2, expressions of which in tissue and also products (superoxide anion, hydrogen peroxide, or both) are different [74]. Some studies suggested that deficiency of NOX1, NOX2 or NOX4 resulted in complete inhibition of AAA, while others suggested that the increase in AAA extent was associated with NOX2 deficiency [84,85]. Jiménez-Altayó et al. [69] provided confirmation of the involvement of NADPH oxidase-dependent ROS in the formation of an aneurysm in Marfan’s syndrome since they demonstrated decreased aneurysm size and ECM breakdown in Fbn1C1039G/+/-Nox4−/− mice [69]. Moreover, they observed higher nitrotyrosine residues in both aortic aneurysms and cultured SMC collected from patients with Marfan’s syndrome [69]. Additionally, direct inhibition of NADPH oxidase was found to diminish MFS aneurysm growth in the Fbn1C1039G/+ MFS mouse model [82]. However, the question concerning the localization of aneurysms solely in the aortic root in MFS patients despite increased TGF-β concentrations throughout the vasculature remains unanswered. Nevertheless, Jiménez-Altayó et al. [69] have identified plausible targets affected by oxidative stress. They suggested the involvement of alpha-smooth muscle actin (α-SMA), encoded by the *ACTA2* gene, since they found that patients with mutations within this gene can develop familial thoracic aortic aneurysms. In the course of aneurysm formation, reactive oxygen species could modulate numerous downstream effectors; for example, they can increase MMP activation and stimulate SMC phenotype switching and apoptosis [81,86]. Enhanced production of ROS in MFS aorta can be triggered by angiotensin II signaling, TGF-β signaling as well as aortic root stress and strain [82]. TGF-β1 directly weakens vasoconstriction, and its overexpression is linked with the upregulation of matrix MMPs [87]. AngII belonging to the renin-angiotensin system stimulates the production of ROS via NADPH oxidase [88]. The finding that AngII locally enhances MFS SMC ROS production in ascending aorta-derived SMC (not in descending aorta-derived SMC), but also in other cell types, including cardiomyocytes, cardiac fibroblasts and vascular SMC, provides confirmation of the aforementioned thesis [89,90]. The second active molecule, TGF-β, can promote oxidative stress either directly or indirectly via AngII. However, the mechanism of AngII action in aneurysm formation may turn out to be different and involve the stimulation of MMP activation [91,92].

Additionally, myeloperoxidase contributes to ROS generation in AAA. According to some studies, the absence of myeloperoxidase or its inhibition hampers AAA formation [93]. Since a product of myeloperoxidase (hypochlorous acid) easily reacts with lipids, it appears that lipid oxidation may be one of the important steps in oxidative damage in the course of AAA [94]. There are also other sources of ROS, such as cyclooxygenase, iNOS, mitochondrial metabolism and xanthine oxidase, which may be involved in the pathogenesis of AAA, however, the impact of the last two seems to be less important [95]. Yang et al. [10] examined various pharmacological inhibitors of superoxide-generating enzymes to identify mechanisms related to increased superoxide levels in the Marfan’s aorta. They found that the inhibition of upregulated xanthine oxidase catalyzes the oxidation of hypoxanthine and xanthine and generates O_2_^−^ and H_2_O_2_, with the use of allopurinol restored contraction and relaxation in the MFS aorta. It has been suggested that upregulated expression of iNOS (which forms excessive NO and ROS) may be the mechanism compensating for diminished NO bioavailability [22,96]. In turn, Soto et al. [97] also found increased activity of glutathione reductase (GR), which restores glutathione (GSH) levels by reducing GSH to oxidized GSH (GSSG), in patients with Loeys-Dietz syndrome (LDS) (a variant of Marfan’s syndrome). However, despite this rise in activity, GSH values were not restored. This finding may be associated with low levels of GSH, which may stimulate the GR activity as well as the alteration of the enzyme γ-glutamyl-cysteine synthase responsible for the GSH synthesis or a deficient amount of the amino acids that constitute GSH [98,99]. Patients with LDS also displayed a low expression of the Nrf2 transcription factor which is responsible for the regulation of inducible expression of some antioxidant enzyme genes, including GPx, SOD isoforms and GST [100]. Therefore, such a decrease in Nrf2 expression could translate into the decreased expression of the aforementioned antioxidants as well as eNOS expression in LDS patients and the aggravation of oxidative stress. Zuñiga et al. [28] showed decreased GSH/GSSG index in MFS patients resulting from alterations in the redox state and leading to disturbances in cellular proliferation, differentiation and death in the aortas of these patients. Many metabolic pathways responsible for the reduction in GSH and an increase in GSSG concentrations could be associated with the progression of the aneurysm in MFS patients. Yang et al. [10] demonstrated that in the mice model of MFS, endothelial dysfunction related to reduced glutathione (GSH) depletion stimulated the progression of thoracic aortic aneurysms. The depletion of GSH results from the accumulation of reactive oxygen species [77]. According to some studies, the inhibition of ROS generation can hamper the formation of an aneurysm in an MFS murine model [101]. In turn, reduction of GPx in MFS patients may be caused by high concentrations of intracellular H_2_O_2_ [28]. However, diminished GPx levels could also be associated with lower selenium in MFS patients since the diminished level of this micronutrient was described in other cardiovascular pathologies; moreover, selenium participates in the regulation of GPx, since it is inserted in enzyme’s active site [102]. Due to the fact that GPx can also reduce ONOO^-^, its reduced activity may lead to the accumulation of reactive nitrogen species in aortic smooth muscle cells of the patients with MFS, which in turn may stimulate aneurysm formation [103,104]. Finally, the diminished GPx activity is related to the ROS-induced activation of the TGF-βR1 receptor, leading to higher TGF-βR1 concentration in the aortic tissue [105]. Patients with Marfan’s syndrome were also found to have decreased GST activity compared to controls. The GST enzyme conjugates GSH to electrophilic xenobiotics, chemicals and toxic compounds, leading to an increase in the rigidity of the cellular membrane [106]. However, oxidative stress inactivates this enzyme [107].

According to some studies, the progression of thoracic aortic aneurysm is the result of the markedly impaired aortic contractile function as well as decreased nitric oxide (NO)-mediated endothelial-dependent relaxation [10,22,108]. ROS impedes calcium signaling, which results in diminished vascular contractility [40,109]. Vasomotor dysfunction, which is strictly regulated by ROS, was suggested to modulate the susceptibility of aneurysm formation [3,110]. The progression of thoracic aortic aneurysm in Marfan’s syndrome is accompanied by significant dysfunction of aortic contractile function [22,111]. The preservation of contraction could protect against aneurysm formation in the abdominal aorta [110]. Chung et al. [22] demonstrated the impairment of NO-mediated endothelium-dependent relaxation in a mouse model of Marfan’s syndrome. Furthermore, they suggested that compromised contractile function found in the MFS thoracic aorta may be the result of the excessive oxidative stress related to the downregulation of SOD as well as the upregulation of superoxide-producing enzymes [22,111,112]. Mouse models of Marfan’s syndrome were found to develop vasomotor dysfunction in the thoracic aorta, which was associated with oxidative stress resulting from the rise in eNOS and reduced production of Mn-SOD and Cu-Zn-SOD [4]. In turn, Soto et al. [5] demonstrated increased activity of Mn-SOD and Cu-Zn-SOD, which could imply the overexpression of MFS patients’ antioxidant system in an attempt to counteract the oxidative stress. According to some studies, the overexpression of the vascular Cu-Zn-SOD protects against reperfusion injury, while cytosolic and extracellular SOD inhibit vascular and myocardial hypertrophy [113,114]. Soto et al. [5] also demonstrated that CAT levels in patients with Marfan’s syndrome were increased. Such an overexpression of CAT may result again from the overproduction of H_2_O_2_ in the aorta of these patients. Pre-incubation of SOD alone or a combination of SOD and catalase was found to normalize the contractile response of Marfan’s aorta to the control level, which confirms the impact of excess superoxide on vasoconstriction in this syndrome [10].

Some studies have shown that senescence is a potential mechanism involved in aneurysm development [115]. Senescence can be described as a cellular state of discontinued cell division, accompanied by a specific inflammatory cytokine profile. Age as well as some stressor factors (DNA damage and oxidative stress) can stimulate its occurrence [116]. The results of animal studies (mice model of MFS) suggested that increased oxidative stress in the aorta of studied animals can potentially act as an inducer of vascular senescence [10].

It is apparent that dysregulation of ROS results in extensive changes in the arterial wall underlying AAA development.

## 5. Oxidative Stress-Reducing Strategies

The identification of key elements of the molecular pathway(s) responsible for aneurysm formation may translate into the development of innovative medical therapies targeted at preventing or slowing aneurysm growth [82,117,118]. The reduction of oxidative stress level has been suggested to potentially improve aortic vasocontractile function and endothelium-dependent relaxation, thus decreasing the susceptibility of aneurysm formation.

### 5.1. Resveratrol

**Figure 1:**
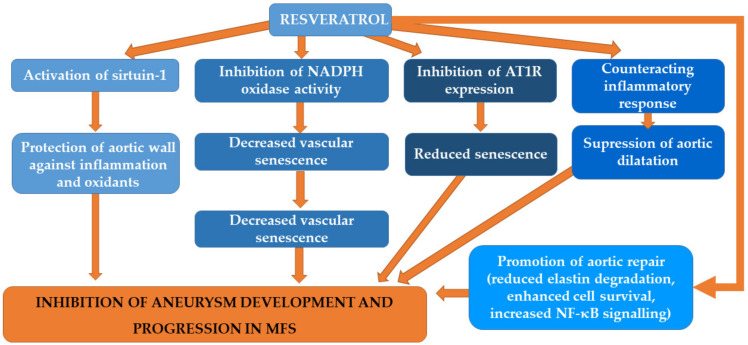
Possible mechanisms underlying the beneficial effects of resveratrol on an aortic aneurysm in MFS.

Resveratrol, which is a biologically potent polyphenol, exerts beneficial effects, including the diminishing of oxidative stress, enhanced calcium handling and the inhibition of pathological hypertrophic signaling [119,120]. Resveratrol not only has antioxidant properties but also activates sirtuin-1 (SirT1) [121]. Sirtuin-1 is a lysine deacetylase participating in the protection of the aortic wall from inflammation and oxidants [121]. Resveratrol was found to decrease vascular senescence via the inhibition of nicotinamide adenine dinucleotide phosphate (NADPH) oxidase activity, and consequently decrease oxidative stress, in a SIRT1-dependent fashion [122]. SIRT1 inhibition markedly enhanced NADPH oxidase activity, vascular superoxide production and mRNA expression of its subunits p22 (phox) and NOX4, and all of these effects were prevented by the administration of resveratrol [123]. The findings that NOX4 is highly increased in the human and murine MFS aorta, and NOX4-deficient MFS mice experience mitigated aortic aneurysm formation and diminished elastin degradation, demonstrate that NOX4 and/or ROS may be involved in the pathogenesis of aortic changes [69]. Resveratrol is also capable of inhibiting the expression of AT1R and limiting its downstream pathways, including increased senescence [122]. Therefore, it has been suggested that resveratrol may influence aneurysm progression in MFS mice [115]. However, it remains unknown whether this is the primary mechanism of action, since the modulation of SIRT1 activity with SRT1720 or sirtinol also altered the senescence, but did not change the aortic root dilatation. Additionally, another study indicated that the administration of resveratrol and losartan to Fbn1C1039G/+ MFS mice inhibited aortic dilatation [115]. Both compounds were demonstrated to diminish aortic senescence. The treatment with resveratrol enhanced the activation of SIRT1 and decreased medial thickening and elastin breaks. However, due to the fact that direct activation or inhibition of SIRT1 failed to exert any impact on aortic root dilatation, Hibender et al. [115] suggested that effects of resveratrol are SIRT1- and senescence-independent. Moreover, they found that resveratrol reduced in vivo and in vitro miR-29b expression in SMCs in an indirect, endothelial cell-dependent manner via increasing NF-κB activity. Furthermore, resveratrol was demonstrated to effectively suppress aortic dilatation by counteracting the inflammatory response [124]. In contrast, Hibender et al. [115] failed to observe the impact of resveratrol on vascular inflammation in MFS mice. These authors suggested that anti-inflammatory drugs decreasing vascular inflammation may have no impact on aortic root dilatation reduction [125]. Apart from the aforementioned activities, the administration of resveratrol was demonstrated to promote aortic repair, which was mirrored by lower elastin degradation, enhanced cell survival (Bcl-2/Mcl-1/less apoptotic cells) and increased NF-κB signaling [115]. Resveratrol was also found to be effective in the case of aortopathy in an MFS mouse model (Fbn1C10393G/+), despite continuous ERK1/2 signaling [120]. Budbazar et al. [121] examined the effect of resveratrol administration (105 mg/kg/day mixed in food) on the mortality of animal models of MFS with aortic aneurysm (fibrillin-1 hypomorphic mice; Fbn1mgR/mgR). They observed that the addition of resveratrol to food was associated with significantly reduced mortality of Fbn1mgR/mgR mice compared to mice on regular chow. The majority of Fbn1mgR/mgR mice died due to aortic rupture/dissection. Based on their observations of considerably decreased plasma levels of active TGF-β1 in resveratrol-fed mice and increased levels of total reversible oxidations of proteins in Fbn1mgR/mgR mice compared to wild-type mice, the authors suggested that resveratrol may diminish the incidence and mortality of aortic aneurysm via reduction of oxidative post-translational modifications of SirT-1 in VSM cells and inhibition of excessive TGF-β1 in Fbn1mgR/mgR mice [121]. Figure 1 summarizes possible mechanisms underlying the beneficial effects of resveratrol on an aortic aneurysm in MFS.

### 5.2. n-3 polyunsaturated fatty acids (PUFAs) (n-3 PUFA): Docosahexaenoic and Lipoic Acids

Recently, some studies have suggested the protective function of heme oxygenase-1 (HO-1) in the abdominal aortic aneurysm (AAA). Heme oxygenase-1 is a stress response protein that plays an essential role in the protection against injury since it possesses antioxidative and anti-inflammatory properties [27]. Its utility has been previously studied in cardiovascular diseases [126,127]. The results of studies carried out on an animal model (haplo-insufficient HO-1 mice) revealed that such animals were more prone to elastase-induced AAA compared to wild-type mice [128]. In turn, Jiang et al. [27] showed that HO-1 expression was critical for AAA growth and severity. Moreover, lack of HO-1 was found to increase the prevalence of AAA and the risk of its rupture in the angiotensin II-induced AAA model [129]. Moreover, higher ROS level-enhanced VSMC apoptosis, significant elastin degradation, macrophage infiltration, as well as MMP activation were also seen in this model [129]. Therefore, many researchers tried to prevent aneurysm formation through the modulation of *HO-1* expression. Meital et al. [130] suggested that n-3 PUFA docosahexaenoic acid (DHA) might provide a therapeutic strategy for AAA. They found that DHA therapy not only suppressed LPS-induced ROS and lowered inflammatory cytokine levels, but also increased HO-1 mRNA levels and glutathione peroxidase activity in macrophages [130]. Additionally, doxycycline, which is a safe and commonly used antibiotic, was found to significantly decrease the incidence and severity of AAA, partly via the upregulation of HO-1 expression [131].

In turn, Guido et al. [132] demonstrated that the treatment of animal models of MFS (mice) with lipoic acid resulted in a significant decrease in ROS production as well as decreased expression of pERK1/2. Lipoic acid is known for its protective effects in several pathological conditions since it can affect ROS-involved signaling cascades, scavenge ROS and restore endogenous antioxidants (e.g., reduced glutathione) [132,133,134]. However, in their study, such therapy was not associated with the progression of aortic remodeling and aneurysm formation. The administration of lipoic acid prevented the prevalence of focal inhomogeneous regions within the aortic arch of MFS animals, and therefore, the authors suggested that oxidative stress may be related to this new process. They also found that losartan markedly hampered aneurysm formation and reduced focal inhomogeneous regions in MFS mice via both ROS-dependent and independent pathways [132].

### 5.3. Hibiscus sabdariffa Linne (HSL)

Recently, *Hibiscus sabdariffa Linne* (HSL), which is used as an antibacterial, antifungal, hypocholesterolemic and cardioprotective agent, has been suggested to be beneficial in patients with Marfan’s syndrome [135,136,137]. This plant contains numerous chemical constituents exerting antioxidant effects, including polyphenols, anthocyanin, flavonoids, L-ascorbic acid and protocatechuic acid (PCA) [135,138]. Wang et al. [139] demonstrated that PCA and catechin contained in HSL could not only scavenge destructive free radicals but also regenerate other antioxidants, preventing cellular oxidative damages. In turn, Soto et al. [137] demonstrated that the activity of the ECSOD enzyme was considerably higher in the control group and MFS patients consuming HSL compared to MFS individuals, which indicates the loss of ECSOD activity with aggravating oxidative stress as well as the protective potential of HSL against such stress. ECSOD, expressed principally on the surface of vascular smooth muscle cells and the subendothelial space, is an antioxidant protecting cells from the potentially detrimental effects of ROS [137]. Furthermore, it was suggested that the diminished activity of ECSOD in MFS could stem from some genetic mutation which also alters vascular function, increasing its susceptibility to aneurysm formation [4]. Other studies have indicated enhanced vascular oxidative stress and modified the endothelium-dependent vasoreactivity following the inhibition of ECSOD [140]. Altered vascular reactivity results from higher levels of free radicals, changing NO metabolism in mice lacking ECSOD [141]. In turn, the deficiency of Cu-Zn-SOD was associated with vascular permeability and ischemia in hypertrophic cerebral arterioles in mice [142]. Reduced activity of ECSOD and GPx isoforms was found to lead to the accumulation of lipid peroxidation (LPO) and subsequent increase in oxidative stress [143].

The administration of HSL may reduce chronic oxidative stress present in MFS patients by enhancing the activity of extracellular GPx [144]. Extracellular GPx, a unique selenium glycoprotein decreasing organic hydroperoxides, hydrogen peroxide and phospholipid hydroperoxides in vitro is involved in the control of ROS-induced oxidative stress in the circulation [145]. Apart from extracellular GPx, HSL calyces also contain anthocyanins, which have anti-inflammatory properties and downregulate tumor necrosis factor α (TNF-α). Soto et al. [137] found markedly decreased activity of GST in the MFS patients when compared to C subjects and MFS + HSL patients. GST is another enzyme involved in the detoxification of ROS [137]. This enzyme catalyzes the conjugation of glutathione with electrophilic compounds, and the resultant conjugation product is actively transported out of the cell [146]. The decrease in GST activity in MFS patients has been confirmed by many studies [5]. Moreover, Soto et al. [137] suggested that reduced GST activity in MFS patients could promote oxidative stress and the accumulation of LPO products, while antioxidant effects of HSL may decrease ROS and LPO product generation, contributing to an increase in GST activity. It appears that the formation of aneurysms and oxidative stress development in MFS could be associated with the build-up of the end products of LPO, including 4-hydroxy-2-trans-nonenal (4-HNE) [137]. High concentrations of 4-HNE stimulate apoptosis in endothelial cells as well as trigger metalloproteinases (MMPs) 1 and 2 activity in vascular smooth muscle cells. Metalloproteinases participate in the degradation of collagen and elastin in the extracellular matrix [147]. Wilton et al. [148] demonstrated the involvement of MMP-2 in the development of aneurysms in the thoracic aorta in MFS. PCA contained in HSL was found to diminish the activity of metalloproteinases [149]. Patients with MFS were also found to have lower glutathione concentration compared to controls and MFS + HSL patients [137]. Glutathione is the most abundant endogenous intracellular antioxidant which inactivates and OH^−^ radicals and transforms vitamins E and C into their active forms [149]. According to studies, GSH plays a vital role in the antioxidant defenses, and its decreased cellular and plasma levels are an indicator of oxidative stress [150]. Insufficient antioxidant defense is associated with the enhanced formation of ONOO^−^ and its accumulation within the cell, leading to cellular oxidative damage [8]. It was demonstrated that GSH can reduce ONOO^−^ [151].

**Figure 2:**
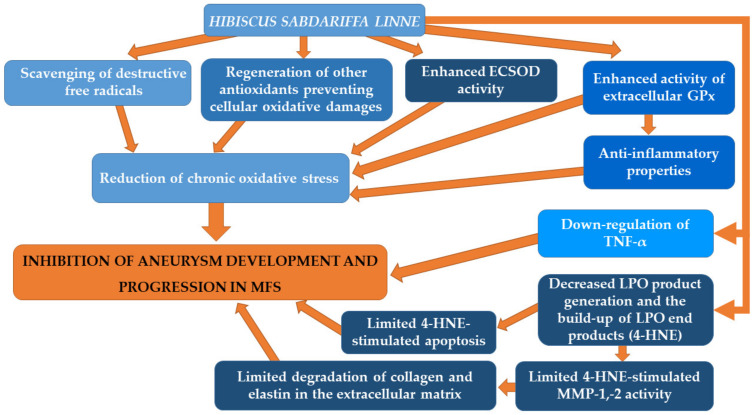
Plausible mechanisms underlying the beneficial effects of *Hibiscus sabdariffa Linne* on aortic aneurysm in MFS.

Figure 2 summarizes plausible mechanisms underlying the beneficial effects of *Hibiscus sabdariffa Linne* on aortic aneurysm in MFS.

### 5.4. Pharmacological Management of MFS

The principal goal of pharmacological therapy in patients with Marfan’s syndrome involves the hampering of the aortic dilatation rate to postpone or prevent complications and surgical interventions [152,153]. Pharmacological therapy of MFS is typically based on ß-adrenoceptor blockade [154]. Beta-blockade was found to decrease the rate of aortic pressure elevations over time as well as to diminish blood pressure alone, thus reducing hemodynamic stress on the proximal aorta [27,64]. Shores et al. [155] suggested that prophylactic beta-adrenergic blockade with propranolol not only reduced the rate of aortic dilatation but also decreased the development of aortic complications. However, the results of other studies are conflicting. Additionally, the use of losartan (angiotensin II receptor blocker (ARB)) was demonstrated to be efficient in MFS mouse experiments, however, in human studies, it did not meet the expectations [156]. Only the COMPARE study found a slight but significant effect of losartan therapy on aortic root dilatation rate in adults with MFS [157]. According to another study, better effects are obtained following combined therapy with a β-blocker and ARB [156,158].

While describing the impact of the aforementioned treatments, we will focus on how they decrease the rate of aortic root dilation via affecting oxidative stress in MFS patients. The results of an animal study (MFS mouse model) indicated that losartan could decrease aortic root widening through the stimulation of protective endothelial function and nitric oxide (NO) bioavailability in the aorta [159]. This mechanism was suggested to be independent of angiotensin II receptor type 1 (ATR1). In turn, Tehrani et al. [160] demonstrated that low-dose valsartan was equally as effective as losartan in enhancing endothelial function and thus correcting endothelial abnormalities and reducing oxidative stress. These results are in agreement with the study revealing that loss of endothelial flow-mediated dilation closely correlated with aortic dilation in MFS patients [161]. Moreover, the use of experimental eNOS-activating peptides, which enhanced eNOS-derived NO release in a redox-independent manner in the descending aorta ex vivo and in vivo, was found to be associated with the inhibition of MFS aortic root widening [159]. Despite the fact that in animal studies (MFS mice), compounds activating endothelial cell function and NO have demonstrated effectiveness, simple stimulation of NO-dependent vasodilatory signaling in SMC may prove to be insufficient to provide aortic stability in patients with protein kinase G1-activating mutation and greater aortic oxidative stress related to a reduced NO bioavailability [58,118,160]. The beneficial impact of ARBs on endothelium, reaching beyond BP-reducing effects, was confirmed in myography and eNOS phosphorylation assessment studies [160]. Since eNOS-specific upregulation of endothelial function can trigger strong anti-remodeling effects, it seems that prophylactic endothelial function-optimized approaches can be beneficial for patients with MFS aortic root disease [159,162].

Additionally, the management of TGF-β signaling and the inhibition of matrix metalloproteinases (MMPs) with the use of doxycycline was found to mitigate TAA progression [163,164,165]. The administration of doxycycline markedly prolonged MFS mice lifespan, delaying aneurysm rupture, possibly as a result of the inhibition of the MMP-2 and MMP-9 production and the subsequent reduction in aortic elastic fiber fragmentation [164,166]. However, such therapy is not directly associated with oxidative stress, and thus it is not the topic of this review.

Finally, it has been suggested that the decrease in the level of mutant *FBN1* transcript can be expected to be an effective therapy for MFS [167]. Hammerhead ribozymes, which are catalytic RNA molecules, can be targeted specifically to FBN1 transcripts in individuals with MFS. As a result, either the downregulation of mutant fibrillin-1 production or complete ablation of endogenous FBN1 expression can be obtained. U1 small nuclear RNA (snRNA) can be utilized as a vehicle for the delivery of a FBN1 hammerhead ribozyme sequence [168]. Indeed, the expression of a chimeric U1 snRNA–ribozyme construct was found to inhibit the expression of fibrillin-1 mRNA and protein in stably transfected cultured cells. The impact of various antioxidant therapies on aneurysm development in MSF has been presented in Table 1. 

## 6. Conclusions

Many studies have reported the involvement of oxidative stress in MFS. Increased oxidative stress in the MFS aortic wall was suggested to be associated with impaired antioxidant regulation in the mutant genotype. Indeed, the activity of antioxidant enzymes (such as SOD isoforms, CAT and GST) was found to be reduced in the TAA of patients with MFS. Lower activities of these enzymes favor ROS production and accumulation, contributing to TAA formation. The results of both animal and human studies demonstrated augmented redox stress and accelerated progression of the aortic aneurysm. In turn, the inhibition of NADPH-oxidase diminished aneurysm formation in a Fbn1C1039G/+ Marfan’s mouse model via decreased MMP activation. Currently, the goal of the treatment of patients with MFS should involve hampering the progression of aortic dilation to avoid catastrophic complications with the use of agents with antioxidant properties.

## Figures and Tables

**Table 1:** Impact of various antioxidant therapies on aneurysm development in MFS.

Type of Therapy	Type of Study	Important Results	Ref.
Resveratrol (Res)	Animal study Adult male Wistar rats fed with HFS diet in the presence or absence of Res for 3 months. Cultured BAECs	Res protected against HFS- or high-glucose-induced increase in NADPH oxidase p47phox expression and decrease in SIRT1 level. Conclusion: Res can reverse the senescence process in the aorta induced by HFS in rats or by exposure to high glucose in cultured BAECs. The underlying mechanism is SIRT1/NADPH oxidase pathway-dependent.	[122]
	Animal study Fbn1 (C1039G/+) MFS mouse model. Cultured SMC	Resveratrol enhanced the nuclear localization of sirtuin-1 in the vessel wall. It had no effect on leukocyte infiltration, activation of SMAD2 and ERK1/2. Resveratrol reduced aortic elastin breaks and decreased micro-RNA-29b expression. Resveratrol’s effect on micro-RNA-29b downregulation was endothelial cell- and nuclear factor κB-dependent.	[115]
	Animal study Male Sprague-Dawley rats on Resveratrol (10 mg/kg/die) or vehicle (Et-OH) alone for 7 days before until 14 days after the AAA induction with elastase	Resveratrol counteracted the CD62L-monocyte subset expansion, CD143 monocyte expression and circulating levels of MMP-9 activity and TNFα associated with AAA induction. Resveratrol markedly attenuated AAA expansion, vessel wall macrophage infiltration and MMP-9, VEGF and TNFα expression, compared with AAA from Et-OH group. Conclusions: Resveratrol limited the monocyte-dependent inflammatory response, macrophage differentiation and aortic lumen enlargement in elastase-induced AAA.	[124]
	Animal study Fibrillin-1 hypomorphic mice; Fbn1mgR/mgR receiving resveratrol (105 mg/kg/day mixed in food)	The addition of resveratrol to food significantly reduced mortality of Fbn1mgR/mgR mice compared to mice on regular chow. Resveratrol-fed mice had considerably decreased plasma levels of active TGF-β1 and higher levels of total reversible oxidations of proteins compared to wild-type mice. Conclusions: Resveratrol may diminish the incidence and mortality of aortic aneurysm via reduction of oxidative post-translational modifications of SirT-1 in VSM cells and inhibition of excessive TGF-β1 in Fbn1mgR/mgR mice.	[121]
Targeted therapies inducing HO-1: heme, rosuvastatin	Porcine pancreatic elastase (PPE) model of AAA induction in HO-1 heterozygous (HO-1^+/−^, HO-1 Het) mice (heme). Murine AAA model (Ang II-ApoE−/−) (resveratrol)	Deficiency in HO-1 leads to augmented AAA development. Peritoneal macrophages from HO-1^+/−^ mice showed increased gene expression of MCP-1, TNF-alpha, IL-1-beta and IL-6, and decreased expression of anti-inflammatory cytokines (IL-10 and TGF-β). Treatment with heme returned AAA progression in HO-1 Het mice to a wild-type profile. Low doses of rosuvastatin can induce HO-1 expression in aortic tissue and suppress AAA progression. Conclusions: Pleiotropic statin effects might be beneficial in AAA, possibly through the upregulation of HO-1.	[128]
n-3 PUFA docosahexaenoic acid (DHA)	Cells obtained from men with small AAA and age-matched male controls incubated with DHA for 1 h before exposure to 0.1 µg/mL LPS for 24 h	DHA supplementation decreased the concentration of TNF-α and IL-6 in macrophage supernatants. DHA increased glutathione peroxidase activity and HO-1 mRNA expression.	[130]
Lipoic acid Losartan	MFS mgΔloxPneo mouse model vs. WT mice: untreated, treated with losartan and treated with lipoic acid	MFS animals treated with lipoic acid showed markedly reduced ROS production and lower ERK1/2 phosphorylation. Aortic dilation and elastic fiber breakdown were unaltered. Absence of focal inhomogeneous regions in MFS animals treated with lipoic acid. Losartan reduced aortic dilation and elastic fiber breakdown despite no change in ROS generation.	[132]
Hibiscus sabdariffa Linne	HSL infusion in MFS patients	Treatment significantly decreased ECSOD (*p* = 0.03), EGPx (*p* = 0.04), GST (*p* = 0.03), GSH (*p* = 0.01) and TAC and ascorbic acid (*p* = 0.02). GSSG-R activity (*p* = 0.04) and LPO (*p* = 0.02) were increased in MFS patients vs. patients receiving the HSL. Conclusions: Infusion of HSL allows an increase in antioxidant capacity of both the enzymatic and nonenzymatic systems, in the plasma of the MSF patients.	[137]
Losartan	MFS mice lacking ATR1a expression	MFS/ATR1a-null mice showed unabated aortic root enlargement and remained fully responsive to losartan. Losartan’s anti-remodeling properties may be ATR- independent. Losartan can activate the endothelial function in mice and patients. In vitro, losartan can increase endothelial NO release in the absence of AngII and correct MFS NO levels in vivo. Conclusions: Increased protective endothelial function, rather than ATR1 inhibition or BP lowering, may be beneficial in preventing aortic root disease in MFS.	[159]
Sub-BP-lowering dose valsartan Hypotensive dose of losartan	Patients with MFS	Valsartan attenuated MFS aortic root widening by 75.9%. A similar effect was seen in the case of a hypotensive dose of losartan (79.4%). Medial thickening, elastic fiber fragmentation and phospho-ERK signaling were inhibited to a similar degree with both treatments. Valsartan and losartan decreased vascular contractility ex vivo in a NO-sensitive fashion. Conclusions: Artic root stability can be achieved with valsartan in absence of BP-lowering effects.	[160]

BAECs—bovine aortic endothelial cells; BP—blood pressure; ERK1/2—extracellular signal-regulated kinase 1/2; Et-OH—ethanol; HFS—high-fat/sucrose; HO-1—heme oxygenase-1; IL-6—interleukin-6; LPS—lipopolysaccharide; SMC—smooth muscle cells; TNF-α—tumor necrosis factor-α; WT—wild-type.

## Data Availability

Not applicable.
